# Exploring the causal relationship between 25-hydroxyvitamin D and asthma: A bidirectional Mendelian randomization study

**DOI:** 10.1097/MD.0000000000044249

**Published:** 2025-08-29

**Authors:** Xiaosheng Wu, Xueqin Zhan, Chao Ouyang

**Affiliations:** a Department of Respiratory and Critical Care Medicine, Nanchang People’s Hospital, Nanchang, Jiangxi Province, PR China; b Nursing Department, Nanchang People’s Hospital, Nanchang, Jiangxi Province, PR China.

**Keywords:** 25-hydroxyvitamin D, asthma, causal relationship, Mendelian randomization

## Abstract

The role of 25-hydroxyvitamin D (25(OH)D) in disease has attracted much attention. However current research findings on the relationship between 25(OH)D and asthma are not completely consistent. This study aims to explore the potential causal relationship between 25(OH)D levels and asthma using a bidirectional Mendelian randomization (MR) approach. Data from large genome-wide association studies related to the study phenotypes were utilized. Genetic correlation data for 25(OH)D were obtained from the European Bioinformatics Institute database, and genetic correlation data for asthma risk were sourced from the FinnGen biobank. A 2-sample MR analysis was conducted, primarily using the inverse-variance weighted method to assess the impact of 25(OH)D levels on asthma. Sensitivity analyses and horizontal pleiotropy tests were performed to ensure the reliability of the results. Additionally, a reverse MR analysis was conducted to explore potential reverse causal relationships. The MR analysis indicated no significant causal relationship between 25(OH)D levels and allergic asthma (odds ratio [OR] = 1.13, 95% CI: 0.91–1.40, *P* = .259) or nonallergic asthma (OR = 1.06, 95% CI: 0.89–1.28, *P* = .501). Furthermore, the reverse MR analysis did not reveal a significant impact of asthma on 25(OH)D levels (allergic asthma: OR = 1.01, 95% CI: 0.99–1.02, *P* = .363; nonallergic asthma: OR = 1.03, 95% CI: 0.98–1.07, *P* = .235). The consistency of results across sensitivity analyses and various statistical methods supports this conclusion. Evidence from bidirectional MR suggests no causal relationship between 25(OH)D levels and either allergic or nonallergic asthma.

## 1. Introduction

Bronchial asthma, commonly known as asthma, is a widespread chronic respiratory disease marked by persistent airway inflammation, hyperresponsiveness, and obstruction. It affects hundreds of millions of people globally and is responsible for an estimated 2,50,000 deaths annually.^[1,2]^ In some regions, the mortality rate is even showing an upward trend, with an all-cause mortality rate as high as 0.95%.^[3]^ Asthma is also a heterogeneous disease, presenting in various phenotypes, including allergic (atopic) and nonallergic (nonatopic) asthma.^[4]^ The etiology of asthma is complex, involving genetic, environmental, nutritional, and immunological factors.^[5,6]^ Despite extensive research, the exact pathogenesis of asthma remains incompletely understood.

Vitamin D is a vital micronutrient for human health, with its active form being 25-hydroxyvitamin D (25(OH)D). It is mainly sourced from sunlight exposure and dietary intake, and has been identified as playing a key role in a wide range of health aspects, beyond just supporting bone health.^[7,8]^ 25(OH)D plays a regulatory role in the immune system, affecting both innate and adaptive immunity. Evidence from observational research suggests that insufficient vitamin D levels may increase susceptibility to various respiratory conditions, including asthma.^[9,10]^ Low serum concentrations of 25(OH)D have been associated with a greater occurrence and severity of asthma, particularly in younger populations such as children and adolescents.^[11]^

However, observational studies are prone to confounding variables and reverse causation, which complicates the ability to establish a direct causal link between 25(OH)D levels and asthma. For example, individuals with asthma may spend less time outdoors, resulting in reduced sunlight exposure and, consequently, lower 25(OH)D levels.^[12,13]^ In this case, the deficiency might be a consequence of asthma rather than its cause. To address these challenges, Mendelian randomization (MR) has gained prominence as an effective epidemiological approach. By using genetic variants as IVs, MR helps mitigate the influence of confounding factors and reverse causation, offering more robust evidence for causal relationships.^[14]^

In this study, we aim to explore the causal relationship between 25(OH)D levels and allergic and nonallergic asthma using a bidirectional MR approach. By leveraging genetic variants linked to 25(OH)D levels as IVs, we aim to determine whether genetically predicted 25(OH)D concentrations influence the likelihood of developing either allergic or nonallergic asthma. Furthermore, we performed a reverse MR analysis to evaluate whether asthma status has an impact on 25(OH)D levels. This comprehensive methodology enables us to investigate potential bidirectional relationships, providing clearer insights into the role of 25(OH)D in asthma development.

## 2. Materials and methods

### 2.1. Study design

**Figure 1.**
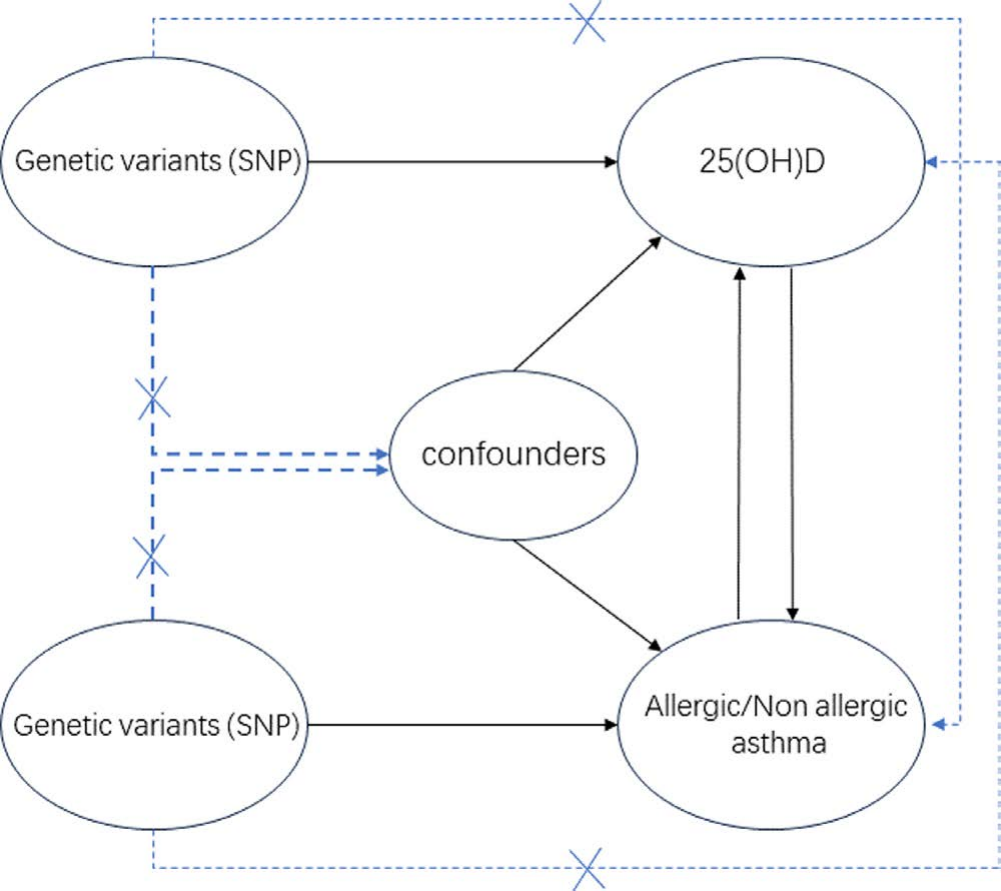
The overview of the MR study design. 25(OH)D = 25-hydroxyvitamin D, MR = Mendelian randomization, SNP = single-nucleotide polymorphism.

This study follows the 3 fundamental assumptions of MR: relevance, independence, and exclusion restriction. In a bidirectional MR framework, genetic variants, specifically single-nucleotide polymorphisms (SNPs), are employed as instrumental variables (IVs) to assess the causal association between 25(OH)D levels and both allergic and nonallergic asthma. The study design is visually represented in Figure 1.

### 2.2. Data sources

The datasets utilized in this analysis were retrieved from publicly accessible databases. The genome-wide association study (GWAS) data concerning 25(OH)D levels were obtained from EBI: ebi-a-GCST90000617 (n = 4,17,580). The GWAS data related to asthma risk were sourced from the FinnGen database: finngen_R9_ALLERG_ASTHMA (n = 2,19,753) and finngen_R11_NONALLERG_ASTHMA_EXMORE (n = 2,47,427). The populations in these 3 datasets come from different countries and years, eliminating the possibility of sample overlap, see Table 1.

**Table 1:** GWAS data sources overview.

Phenotype	Dataset ID	Sample size	Gender	Race	Year	Source
25(OH)D	ebi-a-GCST90000617	4,17,580	Male and female	European	2020	EBI
Allergic asthma	finngen_R9_ALLERG_ASTHMA	2,19,753	Male and female	European	2023	FinnGen
Nonallergic asthma	finngen_R11_NONALLERG_ASTHMA_EXMORE	2,47,427	Male and female	European	2024	FinnGen

25(OH)D = 25-hydroxyvitamin D, GWAS = genome-wide association study.

### 2.3. Selection of instrumental variables

**Figure 2.**
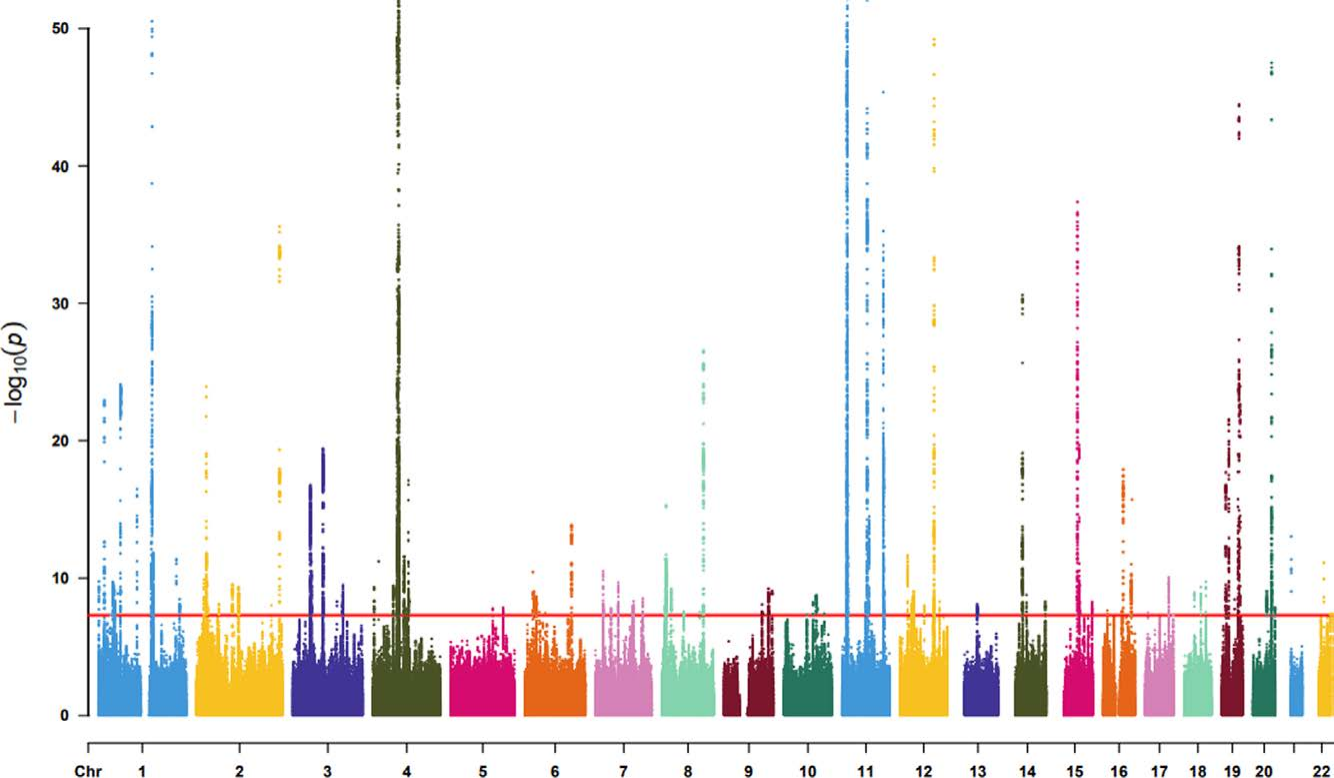
Instrument variables strongly correlated with exposure.

SNPs strongly associated with the exposure factor were selected to ensure their statistical significance (*P* < 5 × 10^−8^), as shown in Figure 2. The independence of these SNPs was assessed to exclude those potentially in linkage disequilibrium. To evaluate the validity of the IVs, the *R*² (coefficient of determination) was calculated using the formula: *R*^2^ = 2 × (1 − MAF) × MAF×β^2^. Additionally, The formula used to calculate the *F*-statistic was *F* = (N − 2) × [*R*²/(1 − *R*²)]. SNPs with an *F*-statistic exceeding 10 were deemed strong IVs for this study.^[15]^

### 2.4. Statistical analysis

The primary analysis employed the inverse-variance weighted (IVW) method, alongside complementary approaches such as MR-Egger, weighted median, and the Wald ratio.^[16,17]^ The IVW method estimates the causal impact of 25(OH)D levels on asthma risk by combining the effects of multiple SNPs using weighted linear regression. A reverse MR analysis was also performed to evaluate whether asthma status influences 25(OH)D levels.

### 2.5. Sensitivity analysis

This study employed several sensitivity analyses, including MR-Egger, the leave-one-out,^[18]^ and the MR-PRESSO approach. Heterogeneity was evaluated using both Egger’s Cochran’s *Q* and IVW Cochran’s *Q* statistics.^[19]^ The MR-Egger intercept served as an indicator of directional pleiotropy, while MR-PRESSO helped detect and remove potential confounders, improving the accuracy of causal effect estimates. In addition, the leave-one-out method systematically excluded each SNP individually, recalculating the results for the remaining SNPs to assess if any one SNP had an undue influence on the association.

### 2.6. Data processing and software

All analyses in this study were carried out using R software (version 4.4.0) in conjunction with the TwoSampleMR and MR-PRESSO packages. Two-sided tests were applied for all analyses, with statistical significance defined as *P* < .05.

## 3. Results

### 3.1. Characteristics of instrumental variables

In the GWAS dataset, SNPs significantly correlated with 25(OH)D levels (*P* < 5 × 10^−8^) were chosen as IVs, while linkage disequilibrium was minimized (*r*² < 0.001, 10,000 kb) and SNPs related to potential confounders were excluded. For allergic asthma and nonallergic asthma, 101 and 102 SNPs were selected, respectively. All chosen IVs had *F*-statistics above 10, effectively ruling out weak instrument bias. In the reverse MR analysis, 18 SNPs linked to allergic asthma and 1 SNP associated with nonallergic asthma were utilized as IVs.

### 3.2. *Causal effect of* 25*(OH)D levels on allergic asthma*

**Figure 3.**
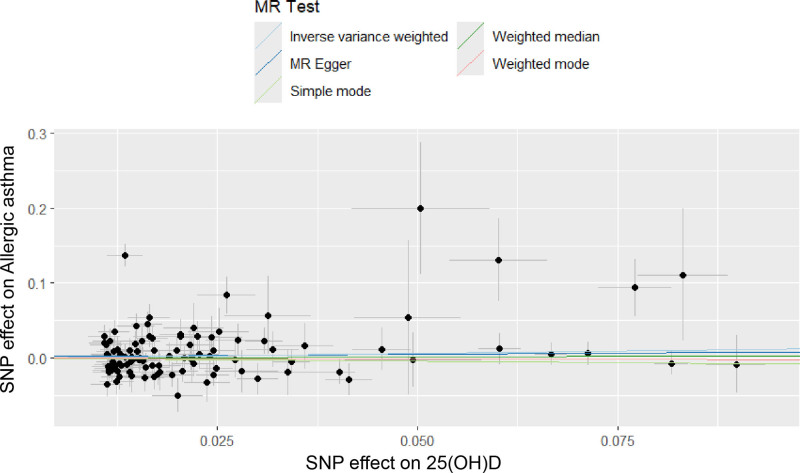
Scatter plot of SNP on “25(OH)D levels and the risk of allergic asthma”. 25(OH)D = 25-hydroxyvitamin D, MR = Mendelian randomization, SNP = single-nucleotide polymorphism.

**Figure 4.**
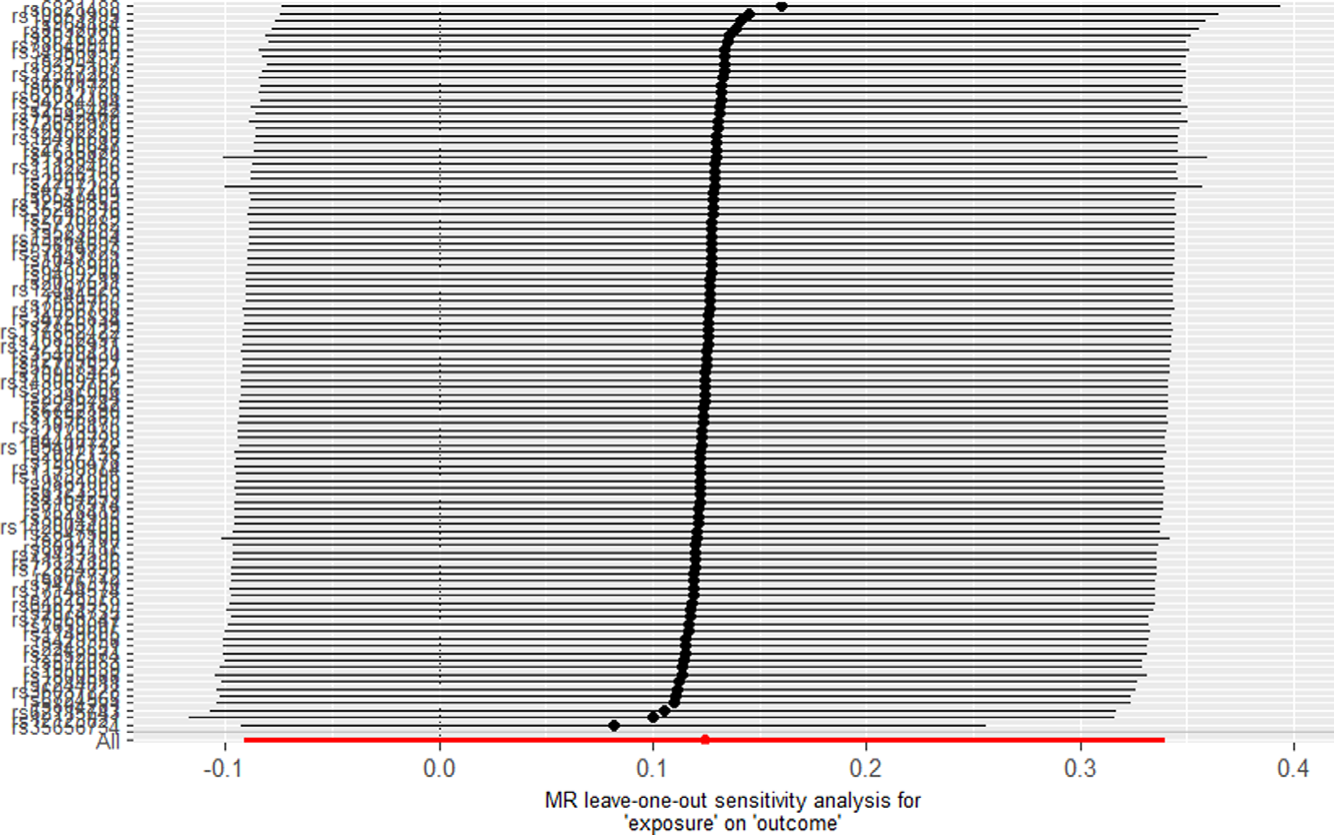
Result of “leave-one-out” sensitivity analysis. MR = Mendelian randomization.

As indicated in Table 2, no significant causal association was found between 25(OH)D levels and the likelihood of developing allergic asthma (odds ratio [OR] = 1.13, 95% CI: 0.91–1.40, *P* = .259). The sensitivity analyses, including scatter plots and leave-one-out methods, yielded consistent findings, as demonstrated in Figures 3 and 4. Furthermore, MR-Egger regression provided no evidence of horizontal pleiotropy (Egger intercept = 0.0019, *P* = .686).

**Table 2:** MR analysis of 25(OH)D levels and the risk of allergic asthma.

Exposure	Outcome	Methods	IVs	OR	95% CI	*P*-value
25(OH)D	Allergic asthma	MR-Egger	101	1.063	0.731–1.546	.749
		Weighted median	101	1.035	0.823-1.302	.768
		Inverse variance weighted	101	1.132	0.913–1.405	.259
		Simple mode	101	0.924	0.593–1.440	.729
		Weighted mode	101	0.984	0.792–1.223	.886

25(OH)D = 25-hydroxyvitamin D, CI = confidence interval, IVs = instrumental variables, MR = Mendelian randomization, OR = odds ratio.

### 3.3. *Causal effect of* 25*(OH)D levels on nonallergic asthma*

**Figure 5.**
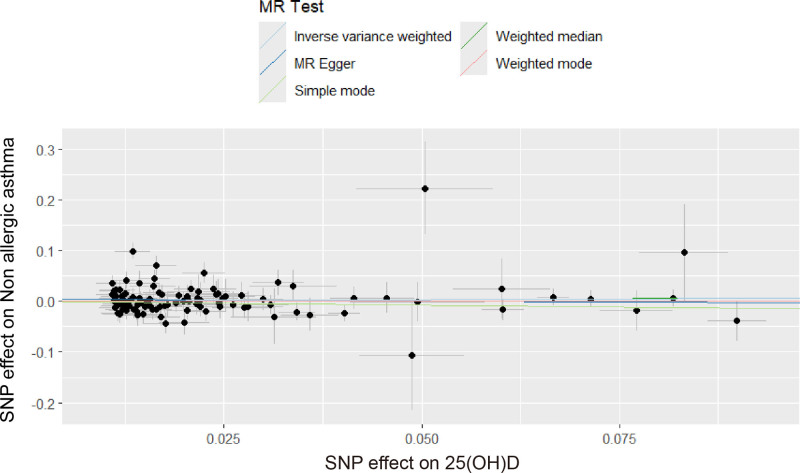
Scatter plot of SNP on “25(OH)D levels and the risk of nonallergic asthma”. 25(OH)D = 25-hydroxyvitamin D, MR = Mendelian randomization, SNP = single-nucleotide polymorphism.

**Figure 6.**
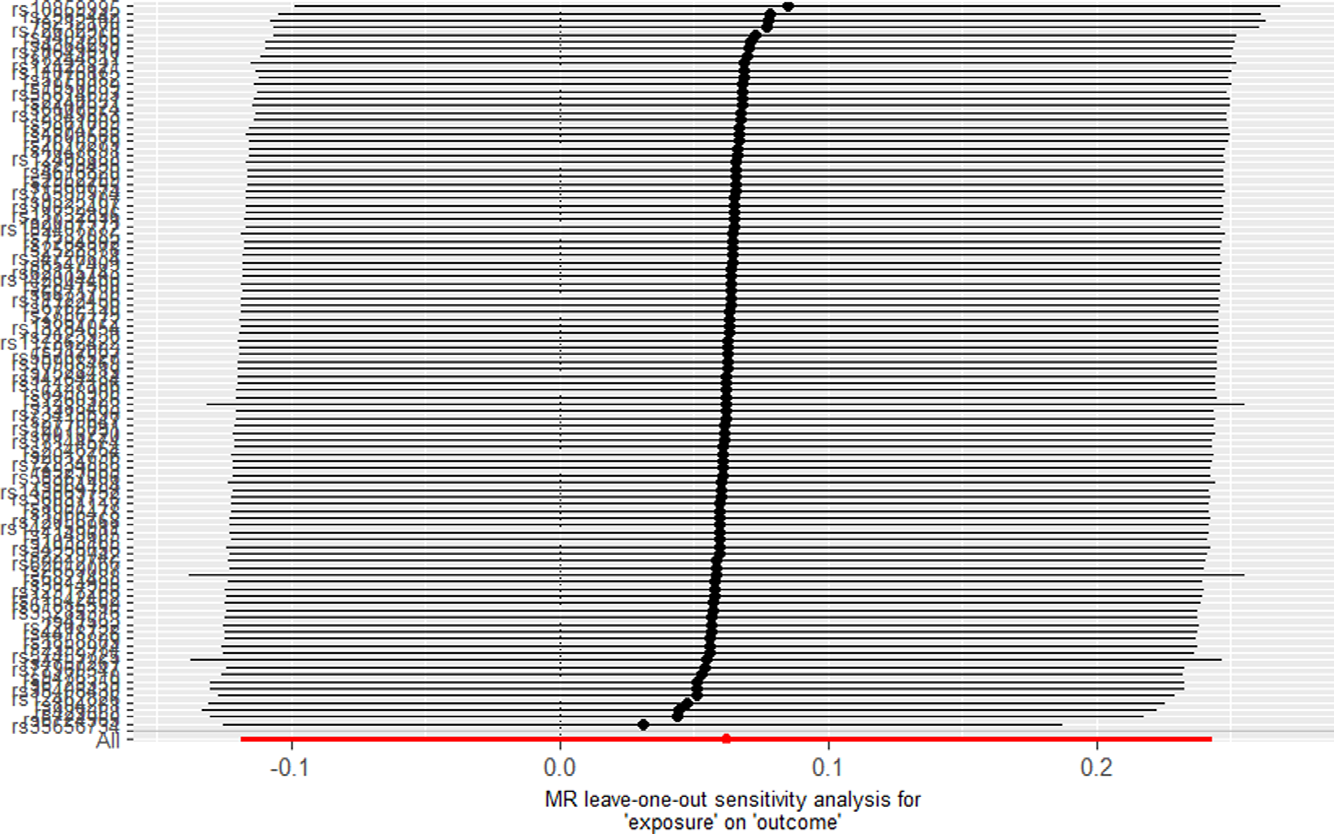
Result of “leave-one-out” sensitivity analysis for 25(OH)D on nonallergic asthma. 25(OH)D = 25-hydroxyvitamin D, MR = Mendelian randomization.

As presented in Table 3, no significant causal link was identified between 25(OH)D levels and the risk of nonallergic asthma (OR = 1.06, 95% CI: 0.89–1.28, *P* = .501). The findings from sensitivity analyses, including scatter plots and leave-one-out assessments, were consistent, as shown in Figures 5 and 6. MR-Egger regression did not indicate horizontal pleiotropy (Egger intercept = 0.0043, *P* = .277), and the MR-PRESSO test also confirmed stable results after removing outliers (*P* = .118).

**Table 3:** MR analysis of 25(OH)D levels and the risk of nonallergic asthma.

Exposure	Outcome	Methods	IVs	OR	95% CI	*P*-value
25(OH)D	Nonallergic asthma	MR-Egger	102	0.923	0.923–0.675	.616
		Weighted median	102	1.071	1.071–0.850	.561
		Inverse variance weighted	102	1.064	1.064–0.888	.501
		Simple mode	102	0.859	0.859–0.559	.491
		Weighted mode	102	1.011	1.011–0.791	.932

25(OH)D = 25-hydroxyvitamin D, CI = confidence interval, IVs = instrumental variables, MR = Mendelian randomization, OR = odds ratio.

### 3.4. Reverse Mendelian randomization analysis

**Figure 7.**
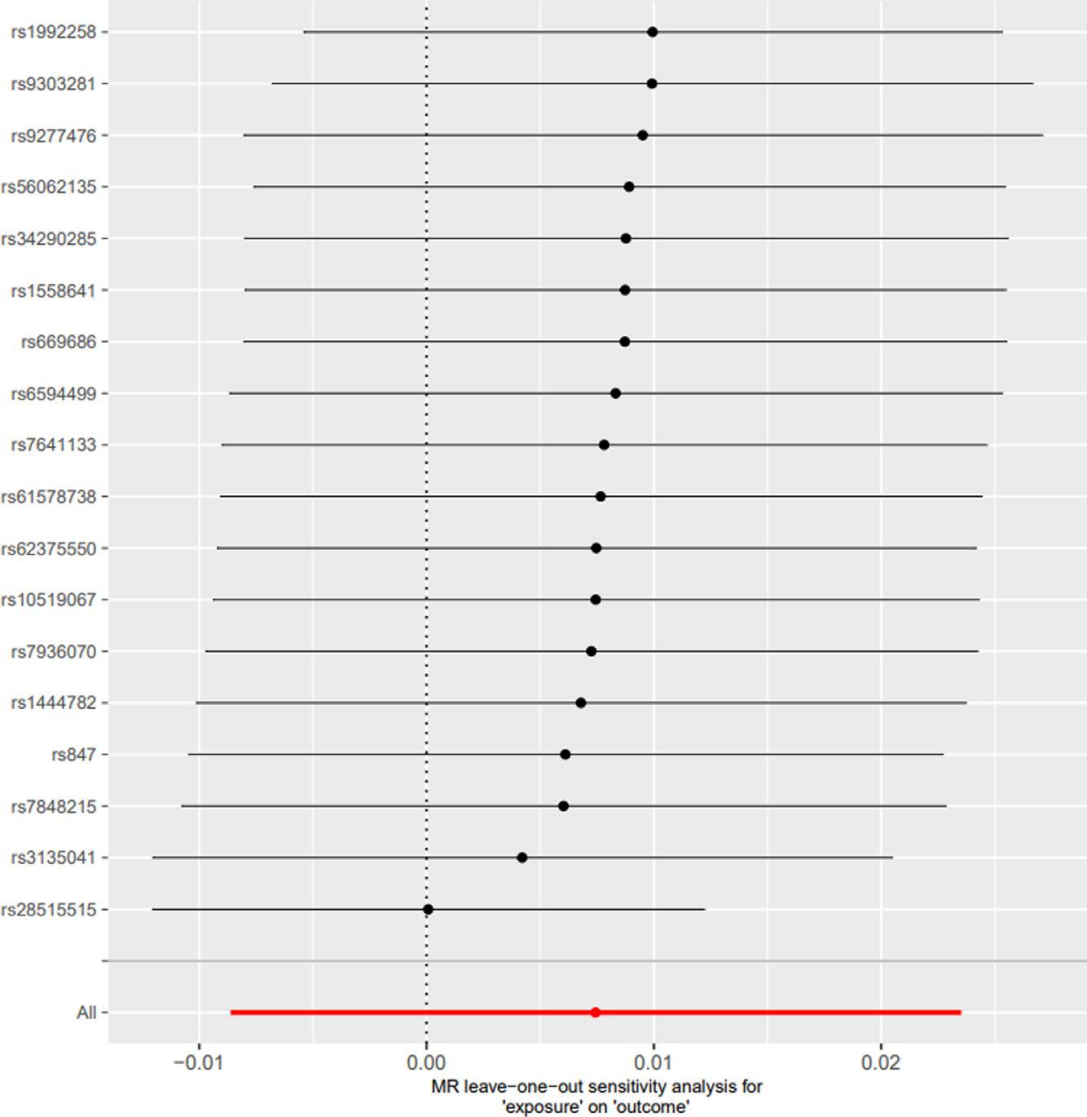
MR “leave-one-out” sensitivity analysis for “allergic asthma” on “25(OH)D”. 25(OH)D = 25-hydroxyvitamin D, MR = Mendelian randomization.

The reverse analysis revealed that allergic asthma had no notable effect on 25(OH)D levels (OR = 1.00, 95% CI: 0.99–1.02, *P* = .36). Sensitivity analyses confirmed these results, with no signs of horizontal pleiotropy (*P* > .05), and the leave-one-out analysis is illustrated in Figure 7. Similarly, nonallergic asthma showed no significant influence on 25(OH)D levels (OR = 1.025, 95% CI: 0.98–1.07, *P* = .23), as detailed in Table 4.

**Table 4:** MR analysis of asthma and 25(OH)D levels.

Exposure	Outcome	Method	nSNP	OR	95% CI	*P*
Allergic asthma	25(OH)D	MR-Egger	18	1.069	0.989–1.156	.111
		Weighted median	18	0.995	0.981–1.008	.428
		Inverse variance weighted	18	1.007	0.991–1.024	.363
		Simple mode	18	0.994	0.972–1.016	.576
		Weighted mode	18	0.991	0.973–1.009	.321
Nonallergic asthma	25(OH)D	Wald ratio	1	1.025	0.984–1.068	.235

25(OH)D = 25-hydroxyvitamin D, CI = confidence interval, MR = Mendelian randomization, OR = odds ratio, SNP = single-nucleotide polymorphism.

## 4. Discussion

This study conducted a comprehensive evaluation of the potential causal link between 25(OH)D levels and both allergic and nonallergic asthma using a bidirectional MR approach. The statistical analyses demonstrated no significant causal association between 25(OH)D levels and either allergic or nonallergic asthma. This outcome may help resolve long-standing ambiguities regarding the connection between 25(OH)D and asthma, carrying important clinical implications.

The findings align with some observational studies,^[20,21]^ but contrast with others,^[22–24]^ which have reported links between 25(OH)D levels and increased asthma risk, worsening symptoms, and reduced lung function. Observational research can be influenced by confounding variables and reverse causality, making those results potentially less reliable. For example, asthma patients might limit outdoor activities due to their condition, leading to reduced sunlight exposure and lower 25(OH)D production.^[25]^ Moreover, factors such as individual diet, socioeconomic status, and health behaviors could affect both 25(OH)D levels and asthma risk.^[26,27]^ As a result, it is difficult to completely account for these confounders in observational studies. The MR approach, which uses genetic variations as IVs, minimizes these confounding influences, offering more reliable causal inferences.^[28]^

In this study, a bidirectional MR design was applied to assess not only the effect of 25(OH)D levels on asthma risk but also the potential impact of asthma status on 25(OH)D levels. The reverse analysis found that asthma status did not significantly influence 25(OH)D levels, thereby ruling out reverse causation and enhancing the validity and robustness of the findings.

Consistency in sensitivity analyses and various statistical methods further support our conclusions. The inverse variance weighted (IVW) method, employed as the primary analysis, aggregated the effects of multiple SNPs using weighted linear regression to estimate the causal relationship between 25(OH)D and asthma risk. Sensitivity analyses, including MR-Egger regression and the weighted median approach, were conducted to ensure the robustness of the findings. Horizontal pleiotropy analysis indicated that the selected SNPs influenced asthma risk only through 25(OH)D levels, not through other pathways, demonstrating the reliability of the IVs and analysis methods.

Although a prior MR study suggested that vitamin D intake might lower asthma risk, it relied on data from a pediatric population and did not differentiate between allergic and nonallergic asthma phenotypes,^[29]^ which may involve distinct etiological mechanisms. Another MR study reported no link between high vitamin D intake and asthma risk,^[30]^ but it similarly did not distinguish between asthma phenotypes and utilized indirect datasets, without directly analyzing data related to 25(OH)D levels. As a result, this study represents the first to directly investigate the causal relationship between 25(OH)D and asthma using the MR approach.

This study delivers robust evidence that convincingly refutes the existence of any causal link between 25(OH)D and either allergic or nonallergic asthma, but there are still some limitations. First, although we selected SNPs significantly linked to 25(OH)D levels as IVs, the variance explained by these SNPs is limited, which may affect the precision of causal effect estimates.^[31]^ Second, despite using various sensitivity analysis methods, it is impossible to completely rule out all potential confounding factors and horizontal pleiotropy. Finally, the outcome data were derived from the FinnGen database, with the study population consisting primarily of individuals of European descent. This could limit the generalizability of the findings, as racial and regional variations may influence the applicability of the results to other populations.

Future research should continue to explore other potential asthma risk factors and further validate these findings. For example, using larger GWAS datasets and multiethnic populations could improve the explanatory power of the IVs and the generalizability of the results. Combining other biomarkers and environmental factors for multifactor MR analysis or conducting large-scale randomized controlled trials could comprehensively evaluate the etiological mechanisms of asthma. Additionally, future studies should focus on other key factors in the 25(OH)D metabolic pathway, Further investigation into components such as the 25(OH)D receptor and the 25(OH)D binding protein is necessary to better understand the potential mechanisms through which 25(OH)D may influence asthma.^[32]^

The findings of this study carry significant implications for public health policy and clinical practice. In light of these results, it is not advisable to recommend vitamin D supplements for the prevention or treatment of asthma. This finding helps avoid unnecessary vitamin D supplementation, reducing potential side effects and economic burden. Public health policies should focus more on other known asthma risk factors, such as air pollution, allergen exposure, and smoking, to develop more effective prevention and intervention measures. In clinical practice, comprehensive management of asthma patients should be emphasized, considering the combined effects of multiple factors rather than relying solely on single nutrient supplementation.

## 5. Conclusion

Evidence from bidirectional MR indicates that there is no significant causal relationship between 25(OH)D levels and either allergic or nonallergic asthma. This suggests that vitamin D supplementation should not be recommended as a means to prevent or treat asthma.

## Author contributions

**Conceptualization:** Xiaosheng Wu.

**Data curation:** Xiaosheng Wu, Chao Ouyang.

**Formal analysis:** Xiaosheng Wu, Xueqin Zhan, Chao Ouyang.

**Methodology:** Xiaosheng Wu.

**Project administration:** Xueqin Zhan.

**Software:** Chao Ouyang.

**Validation:** Xiaosheng Wu.

**Visualization:** Xueqin Zhan.

**Writing** – **original draft:** Xueqin Zhan, Chao Ouyang.

**Writing** – **review & editing:** Xiaosheng Wu.

## References

[1] Venkatesan P. 2023 GINA report for asthma. Lancet Respir Med. 2023;11:589. 37302397 10.1016/S2213-2600(23)00230-8

[2] Rebuck AS. The global decline in asthma death rates: can we relax now? Asia Pac Allergy. 2013;3:200. 23956967 10.5415/apallergy.2013.3.3.200 PMC3736367

[3] Engelkes M de Ridder MA Svensson E. Multinational cohort study of mortality in patients with asthma and severe asthma. Respir Med. 2020;165:105919. 32174450 10.1016/j.rmed.2020.105919

[4] Mims JW. Asthma: definitions and pathophysiology. Int Forum Allergy Rhinol. 2015;5(S1):S2–6. 26335832 10.1002/alr.21609

[5] Mukherjee AB Zhang Z. Allergic asthma: influence of genetic and environmental factors. J Biol Chem. 2011;286:32883–9. 21799018 10.1074/jbc.R110.197046 PMC3190897

[6] Yaari E Susser Z Lahad A. Genetic-environmental interactions in asthma and allergy: a study in a closed population exposed to different environments. Ann Allergy Asthma Immunol. 2009;102:233–7. 19354070 10.1016/S1081-1206(10)60086-5

[7] Brustad M Meyer HE. Vitamin D – a scoping review for Nordic nutrition recommendations 2023. Food Nutr Res. 2023;67:10230. 10.29219/fnr.v67.10230 PMC10710863 38084153

[8] Mahmoud Fawzi M Farouq Fadel Alomari O. Rickets in offspring delivered to vitamin D deficient mother: a review of literatures. Ann Coll Med Mosul. 2023;45:237–46.

[9] Gaudet M Plesa M Mogas A Jalaleddine N Hamid Q Al Heialy S. Recent advances in vitamin D implications in chronic respiratory diseases. Respir Res. 2022;23:252. 36117182 10.1186/s12931-022-02147-x PMC9483459

[10] Goyal JP. Vitamin D and respiratory diseases. Indian J Pediatr. 2024;91:67–72. 37945979 10.1007/s12098-023-04904-2

[11] Alyasin S Momen T Kashef S Alipour A Amin R. The relationship between serum 25 hydroxy vitamin D levels and asthma in children. Allergy Asthma Immunol Res. 2011;3:251–5. 21966605 10.4168/aair.2011.3.4.251 PMC3178823

[12] Rance K. The emerging role of vitamin D in asthma management. J Am Assoc Nurse Pract. 2014;26:263–7. 24170480 10.1002/2327-6924.12062

[13] Jolliffe DA Stefanidis C Wang Z. Vitamin D metabolism is dysregulated in asthma and chronic obstructive pulmonary disease. Am J Respir Crit Care Med. 2020;202:371–82. 32186892 10.1164/rccm.201909-1867OC PMC7397796

[14] Hu X Cai M Xiao J. Benchmarking Mendelian Randomization methods for causal inference using genome-wide association study summary statistics. Am J Hum Genet. 2024;111:1717–35. 39059387 10.1016/j.ajhg.2024.06.016 PMC11339627

[15] Burgess S Thompson SG; CRP CHD Genetics Collaboration. Avoiding bias from weak instruments in Mendelian randomization studies. Int J Epidemiol. 2011;40:755–64. 21414999 10.1093/ije/dyr036

[16] Slob EAW Burgess S. A comparison of robust Mendelian randomization methods using summary data. Genet Epidemiol. 2020;44:313–29. 32249995 10.1002/gepi.22295 PMC7317850

[17] Bowden J Davey Smith G Burgess S. Mendelian randomization with invalid instruments: effect estimation and bias detection through Egger regression. Int J Epidemiol. 2015;44:512–25. 26050253 10.1093/ije/dyv080 PMC4469799

[18] Burgess S Bowden J Fall T Ingelsson E Thompson SG. Sensitivity analyses for robust causal inference from Mendelian randomization analyses with multiple genetic variants. Epidemiology. 2017;28:30–42. 27749700 10.1097/EDE.0000000000000559 PMC5133381

[19] Greco M FD Minelli C Sheehan NA Thompson JR. Detecting pleiotropy in Mendelian randomisation studies with summary data and a continuous outcome. Stat Med. 2015;34:2926–40. 25950993 10.1002/sim.6522

[20] Sung M. Trends of vitamin D in asthma in the pediatric population for two decades.pdf. 2023. https://www.e-cep.org/upload/pdf/cep-2022-01109.pdf. Accessed August 19, 2024. 10.3345/cep.2022.01109 PMC10397993 37321572

[21] Mehrabi S Toghraee E. Association between serum 25-hydroxy vitamin D levels and severity of asthma. Clin Nutr ESPEN. 2022;49:197–200. 35623813 10.1016/j.clnesp.2022.04.025

[22] Sowjanya DS Ravindranath M. Association between Vitamin D deficiency and lung function in asthma patients. IP Indian J Immunol Respir Med. 2023;6:156–60.

[23] Alkhatatbeh MJ Almomani HS Abdul-Razzak KK Samrah S. Association of asthma with low serum vitamin D and its related musculoskeletal and psychological symptoms in adults: a case-control study. NPJ Prim Care Respir Med. 2021;31:27. 33990605 10.1038/s41533-021-00239-7 PMC8121852

[24] Chang Q Zhu Y Zhou G. Vitamin D status, sleep patterns, genetic susceptibility, and the risk of incident adult-onset asthma: a large prospective cohort study. Front Nutr. 2023;10:1222499. 37457981 10.3389/fnut.2023.1222499 PMC10349527

[25] Bener A Ehlayel MS Tulic MK Hamid Q. Vitamin D deficiency as a strong predictor of asthma in children. Int Arch Allergy Immunol. 2012;157:168–75. 21986034 10.1159/000323941

[26] Lo CCH Ng DKK. Vitamin D deficiency and its impact on respiratory health in the Hong Kong pediatric population: current evidence and future directions. Pediatr Respir Crit Care Med. 2023;7:43–9.

[27] Aziz DA Abbas A Viquar W Munawar Hussain A. Association of vitamin D levels and asthma exacerbations in children and.pdf. 2023. https://www.monaldi-archives.org/macd/article/download/2230/1592. Accessed July 31, 2024. 10.4081/monaldi.2022.2230 35608518

[28] Wang Y Shen H. Factors influencing the application of Mendelian randomization studies in causal inference and the challenges in interpreting their results. Chin J Epidemiol. 2020;41:1231–6. 10.3760/cma.j.cn112338-20200521-00749 32867428

[29] Luo L Chen G Zhou Y Xiang Y Peng J. Dietary intake, antioxidants, minerals and vitamins in relation to childhood asthma: a Mendelian randomization study. Front Nutr. 2024;11:1401181. 10.3389/fnut.2024.1401881 PMC11153797 38846540

[30] Yang W Yang Y He L. Dietary factors and risk for asthma: a mendelian randomization analysis. Front Immunol. 2023;14:1126457. 36911739 10.3389/fimmu.2023.1126457 PMC9992976

[31] Jiao R Li W Song J Chen Z. Causal association between asthma and periodontitis: a two-sample Mendelian randomization analysis. Oral Dis. 2024;30:1564–72. 36959704 10.1111/odi.14565

[32] Tapia G Mårild K Dahl SR. Maternal and newborn vitamin D-binding protein, vitamin D levels, vitamin D receptor genotype, and childhood type 1 diabetes. Diabetes Care. 2019;42:553–9. 30692241 10.2337/dc18-2176 PMC6905492

